# Anti-Inflammatory, Barrier-Protective, and Antiwrinkle Properties of *Agastache rugosa* Kuntze in Human Epidermal Keratinocytes

**DOI:** 10.1155/2020/1759067

**Published:** 2020-10-26

**Authors:** Yoonjin Lee, Hye-Won Lim, In Wang Ryu, Yu-Hua Huang, Minsik Park, Young Min Chi, Chang-Jin Lim

**Affiliations:** ^1^College of Life Sciences and Biotechnology, Korea University, Seoul 02841, Republic of Korea; ^2^R & D Center, Shebah Biotech Inc., G-Tech Village, Chuncheon 24398, Republic of Korea; ^3^Institute of Liberal Education, Kangwon National University, Samcheok 25913, Republic of Korea; ^4^Departments of Molecular and Cellular Biochemistry, Kangwon National University School of Medicine, Chuncheon 24341, Republic of Korea; ^5^Department of Biochemistry, Kangwon National University, Chuncheon 24341, Republic of Korea

## Abstract

This work aimed to assess the skin-beneficial properties of *Agastache rugosa* Kuntze, an herbal medication used to treat different types of disorders in traditional folk medicine. The total phenolic compounds and total antiradical, nitrite scavenging, superoxide scavenging, antielastase, and antihyaluronidase activities of a hot water extract of *A. rugosa* Kuntze leaves (ARE) were spectrophotometrically determined. Intracellular reactive oxygen species (ROS) was fluorometrically quantitated using 2′,7′-dichlorodihydrofluorescein diacetate (DCFH-DA). Inducible nitric oxide synthase (iNOS) and filaggrin were evaluated using Western analysis. Real-time quantitative RT-PCR was used to measure filaggrin mRNA. Caspase-14 activity was determined using a fluorogenic substrate. ARE contained the total phenolic content of 38.9 mg gallic acid equivalent/g extract and exhibited 2,2′-diphenyl-1-picrylhydrazyl (DPPH) radical, superoxide radical, and nitrite scavenging activities with the SC_50_ values of 2.9, 1.4, and 1.7 mg/mL, respectively. ARE exerted suppressive activities on nitric oxide (NO) and ROS levels elevated by lipopolysaccharide (LPS) or tumor necrosis factor-*α* (TNF-*α*) in HaCaT keratinocytes. It attenuated the LPS-stimulated expression of iNOS. ARE augmented the UV-B-reduced filaggrin expression on both protein and mRNA levels and was capable of upregulating the UV-B-reduced caspase-14 activity. ARE inhibited *in vitro* elastase and hyaluronidase activities associated with the wrinkling process. ARE, at the concentrations used, did not interfere with the viability of HaCaT keratinocytes. These findings preliminarily imply that the leaves of *A. rugosa* possess desirable cosmetic potentials, such as anti-inflammatory, barrier protective, and antiwrinkle activities, which infers their skin healing potentials.

## 1. Introduction

The skin protects against the penetration of noxious agents, such as allergens, irritants, and microbes, as well as against excessive transepidermal water loss. Impairment of this so-called “skin barrier function” causes environmental allergens to penetrate easily into the skin and to trigger immunological reactions and inflammation, leading to eczema, especially contact dermatitis and atopic dermatitis (AD) [1]. Skin barrier function undergoes undesirable alterations in diseased skin conditions such as senile xerosis and AD and during aging and other adverse conditions, including UV irradiation [2].

The anucleated stratum corneum (SC), mainly responsible for skin barrier function, consists of a multilayer tissue composed of flattened cornified cells or corneocytes containing cytoplasmic proteins, such as keratins, and their degradation products, including natural moisturizing factors (NMFs), cornified cell envelope (CE), cornified lipid envelope, and intercellular lipid layers containing enriched ceramides, cholesterol, and free fatty acids [3]. Skin diseases such as AD and psoriasis and aged skin are characterized by the reduced levels of ceramides that are associated with dysfunctional skin barrier and dryness [4].

The CE, generated during the late stages of epidermal differentiation, is essential for barrier function of the SC. The CE is formed by the assembly of CE precursor proteins, such as filaggrin, involucrin, loricrin, and other small proline-rich proteins, which are covalently cross-linked by epidermal transglutaminases. Filaggrin is required for the retention of water in the SC, and deficient filaggrin results in the reduced NMF components of the SC and the consequent dysfunction of skin barrier [5]. Involucrin acts as a scaffold to which other proteins become cross-linked and resides to the cell membrane and forms the exterior surface of the CE [6]. Loricrin, comprising about 80% of the total protein mass of the CE, acts as a main reinforcement protein for the CE and is deposited onto a scaffold of involucrin and other calcium-binding proteins [7]. Expression of involucrin is attenuated in both acute lesional and nonlesional skin of AD subjects, as compared to the skin from healthy subjects [8].

Caspase-14, activated during keratinocyte cornification, is a cysteine-aspartic acid protease involved in the degradation of filaggrin into free amino acids, some of which contribute to the production of NMFs [9]. Caspase-14-deficient mice display diminished epidermal barrier function and enhanced sensitivity to UV-B radiation, and the defective filaggrin degradation in caspase-14-deficient skin gives rise to a substantial reduction in the amount of NMFs, such as urocanic acid and pyrrolidone carboxylic acid [10].

Wrinkle formation is a complex process that involves various extrinsic factors, such as UV irradiation, environmental pollution, and excessive alcohol consumption, as well as age-dependent decline of skin cell function [11]. It is linked with an attenuation in collagen content that determines the elasticity of the skin tissue [11]. Wrinkles, also as a striking feature of skin photoaging, are associated with oxidative stress and inflammatory response [12].

Since hyaluronidase catalyzes the hydrolytic degradation of hyaluronic acid (HA), its inhibition is considered to hinder skin aging. An extract of *Tagetes erecta* flowers, traditionally used to treat skin diseases, shows effective inhibitory activities on hyaluronidase, elastase, and matrix metalloproteinase (MMP)-1, which implies its antiwrinkle property [13]. Elastase breaks down elastin in an elastic fiber that, together with collagen, determines the mechanical properties of connective tissue. Skin fibroblast-derived elastase has a great impact in affecting the three-dimensional configuration of elastic fiber network, whose elevated activity, elicited by UV irradiation, deteriorates the three-dimensional straight architecture, which in turn results in diminished skin elastic properties [14]. Determination of the enzymatic and molecular properties of skin fibroblast-derived elastase can be used to prevent or protect UV-exposed and aged skin from wrinkling or sagging [14].

*Agastache rugosa* Kuntze (Lamiaceae), a perennial herb grown throughout East Asian Countries, including Korea, Japan, and China, has been used to treat colds, anorexia, cholera, vomiting, miasma, and so on in traditional folk medicine [15]. Its diversified pharmacological properties, such as antimicrobial, antifungal, insecticidal, antiviral, antihypertensive, anti-inflammatory, anticancer, antioxidant, antiatherogenic, and vasorelaxant activities, have been recognized [16–20]. Acacetin, purified from *A. rugosa* leaves, and its derivative acacetin 7-*O*-(6-*O*-malonylglucoside) inhibit monoamine oxidase A and B, suggesting their potentials as lead compounds for inhibitor development [21]. Demethyleugenol *β*-D-glucopyranoside of *A. rugosa* exhibits antimelanogenic effect in four different experimental models, such as melan-a mouse melanocytes, normal human epidermal melanocytes, zebra fish, and reconstructed skin tissue models, through the downregulation of sex-determining region Y-related high-mobility group box 9 and microphthalmia-associated transcription factor [22].

The skin-beneficial potentials of *A*. *rugosa* leaves have been further evaluated during the recent years. ARE plays a protective role against ultraviolet-B- (UV-B) induced photoaging through the downregulation of UV-B-induced ROS, proMMP-2, and -9 in HaCaT keratinocytes, presumably based upon the upregulation of antioxidant components, such as glutathione (GSH) and superoxide dismutase (SOD), diminished under UV-B irradiation [23]. When ARE was subjected to the probiotic bacterial fermentation using *Lactobacillus rhamnosus* HK-9, the fermented ARE was shown to possess higher antioxidant and anti-inflammatory activities than does ARE in LPS-stimulated HaCaT keratinocytes [24]. Similarly, the fermented ARE appeared to contain higher attenuating activity on the UV-B-induced ROS, proMMP-2, and -9, and higher augmenting activity on the UV-B-reduced total GSH and SOD in HaCaT keratinocytes, compared to ARE [25]. These findings suggest that probiotic bacterial fermentation would be used as a tool for improving some therapeutic and cosmetic values of *A. rugose* leaves.

Since we became interested in the dermatological benefits of *A. rugosa* leaves chosen as a candidate resource for the manufacture of improved functional cosmetics, their aqueous extract was previously assessed to have some skin beneficial properties, such as a protective activity against UV-B-induced photoaging, an upregulating activity on some antioxidant components and an antioxidant activity. In this work, we have focused on the barrier protective in the human epidermal keratinocyte cell line HaCaT and *in vitro* antiwrinkle properties of *A. rugosa* leaves, and that we tried to further confirm the anti-inflammatory activity of the leaves using varied experimental protocols.

## 2. Materials and Methods

### 2.1. Reagents

Ascorbic acid (AA), bovine serum albumin (BSA), Folin-Ciocalteu reagent, 2,2′-diphenyl-1-picrylhydrazyl (DPPH), sodium nitrite, Bradford reagent, 3-(4,5-dimethylthiazol-2-yl)-2,5-diphenyltetrazolium bromide (MTT), 2′,7′-dichlorodihydrofluorescein diacetate (DCFH-DA), lipopolysaccharide (LPS), tumor necrosis factor-*α* (TNF-*α*), gallic acid, NADH, nitroblue tetrazolium, phenazine methosulfate, CHAPS, dithiothreitol, *N*-succinyl-(L-Ala)_3_-*p*-nitroanilide, elastase, hyaluronidase, hyaluronic acid (HA), epigallocatechin gallate (EGCG), apigenin, and Griess reagent were from Sigma-Aldrich Chemical Co. (St Louis, MO, USA). Ac-WEHD-methyl-coumarin amide (Ac-WEHD-MCA) was from Peptide Institute Inc. (Osaka, Japan). Cell lysis buffer [25 mM Tris-phosphate (pH 7.8), 2 mM 1,2-diaminocyclohexane-N,N,N′,N′-tetraacetic acid, 2 mM dithiothreitol, 10% glycerol, 1% Triton X-100] was obtained from Promega Korea (Seoul, Korea). All other chemicals used in this work were of the highest grade commercially available.

### 2.2. Source of Plant Material

Dried *A. rugosa* leaves, purchased from a local market, Chuncheon, Korea, in September 2015, were authenticated by Prof. Ki-Oug Yoo, Department of Biological Sciences, Kangwon National University, Chuncheon, Korea. The voucher specimen was deposited in the herbarium of the Department of Biological Sciences, Kangwon National University under the acquisition number KWNU90446.

### 2.3. Extraction

As previously described [23], the dried leaves, ground under liquid nitrogen and mixed with 10-fold distilled water, were extracted under reflux by placing in a water bath at 90°C for 4 h. After being chilled and filtered through a filter paper, the hot water extract was evaporated to dryness in a freeze dryer, and the extract powder was named as ARE. The yield was 10.4%. ARE was dissolved in dimethyl sulfoxide, and control cells were treated with vehicle only (0.1% dimethyl sulfoxide). The vehicle used was convinced to have no effect on cellular viability.

### 2.4. Quantitation of Total Phenolic Compounds

Total phenolic compounds in ARE were quantitated using Folin-Ciocalteu method [26]. The reaction mixture containing 10 *μ*L ARE solution, 90 *μ*L distilled water, and 10 *μ*L of 1 N Folin-Ciocalteu reagent was allowed to stand for 5 min at room temperature. After 100 *μ*L of 7% Na_2_CO_3_ was added to the mixture, the total mixture was kept at room temperature for further 10 min, and the absorbance was measured at 750 nm using a microplate reader (Molecular Devices, Sunnyvale, CA, USA). The calibration curve was set up using gallic acid (0–1 mg/mL), and the index of total phenolic compounds was expressed as mg gallic acid equivalent per g extract.

### 2.5. Antiradical Activity Assay

The total antiradical activity was detected according to the previously described DPPH radical scavenging activity assay [27]. The reaction mixture consisting of 30 *μ*L ARE solution at the concentrations of 0.5, 1, 2, and 4 mg/mL and 270 *μ*L of 0.1 mM DPPH was incubated in the dark at room temperature for 30 min, and the absorbance was measured at 517 nm using a microplate reader. AA was used as a positive control, and the ARE concentration eliciting 50% scavenging of DPPH radicals (SC_50_) was calculated.

### 2.6. Superoxide Radical Scavenging Activity Assay

As previously described [28], the superoxide radical scavenging activity of ARE was determined. ARE solution (20 *μ*L), at the concentrations of 0.5, 1, 2, and 4 mg/mL, was mixed with 180 *μ*L of 1 mM Tris buffer (pH 8.0) containing 312 *μ*M NADH, 200 *μ*M nitroblue tetrazolium, and 40 *μ*M phenazine methosulfate. The absorbance at 560 nm was determined using a microplate reader. AA was used as a positive control.

### 2.7. Nitrite Scavenging Activity Assay

The nitrite scavenging activity was determined as previously described [29]. ARE solution (60 *μ*L), at the concentrations of 0.5, 1, 2, and 4 mg/mL, was mixed with 30 *μ*L of 0.1 M citrate buffer (pH 3.0) and 6.0 *μ*L of 50 *μ*g/mL NaNO_2_. After distilled water was added up to 150 *μ*L, the mixture was immediately incubated for 60 min at 37°C. The 150 *μ*L of Griess reagent (1% sulfanilic acid in 35% acetic acid and the equal volume of 0.1% *N*-1-naphthyl-ethylenediamine dihydrochloride in distilled water) was then added to the mixture, and the absorbance at 550 nm after 10 min was measured using a microplate reader. AA was used as a positive control.

### 2.8. Cell Growth

An immortalized HaCaT keratinocyte cell line (ATCC, Manassas, VA, USA) was cultured in DMEM with 10% heat-inactivated FBS, 100 U/mL penicillin, and 100 *μ*g/mL streptomycin in a humidified atmosphere with 5% CO_2_ at 37°C.

### 2.9. UV-B Irradiation

As an UV-B source, an ultraviolet lamp (peak, 312 nm; model VL-6 M, Vilber Lourmat, Marine, France) was utilized with a radiometer (model VLX-3 W, Vilber Lourmat, Marine, France) equipped with a sensor (bandwidth, 280 to 320 nm; model CX-312, Vilber Lourmat, Marine, France). HaCaT cells were subjected to the solar-simulated UV-B radiation at 70 mJ/cm^2^, the intensity of which induces an oxidative stress without influencing the cell viability.

### 2.10. Cellular Lysate Preparation

Adherent cells were two times washed with phosphate-buffered saline (PBS) and stood on ice for 5 min. The cells were taken out using a cell scraper. After centrifugation at 5,000 g for 10 min, the cell pellets were resuspended in cell lysis buffer and stood for 30 min on ice. Cellular lysate was taken after centrifugation at 5,000 g for 15 min.

Protein contents in cellular lysates were determined according to Bradford protein assay [30] using BSA as a reference protein.

### 2.11. Cellular Viability Assay

As previously described [31], cells were subjected to ARE for 1 h, and then washed out. After the medium was suctioned out, the cells were treated with 5 *μ*g/mL MTT for 4 h. The purple formazan crystals were dissolved in dimethyl sulfoxide. The amount of formazan was determined by the absorbance at 540 nm.

### 2.12. Quantitation of Nitrite in Conditioned Medium

Accumulated nitrite (NO_2_^−^) in conditioned medium was similarly quantitated based on the procedure described in “Section 2.7.” An equal volume of Griess reagent was incubated with conditioned medium for 10 min at room temperature, and the absorbance at 550 nm was measured using a microplate reader. The calibration curve was constructed using known concentrations (0–160 *μ*M) of sodium nitrite.

### 2.13. Quantitation of Intracellular ROS

A redox-sensitive fluorescent probe DCFH-DA, which produces the fluorescent 2′,7′-dichlorofluorescein (DCF; *λ*_excitation_ = 485 nm, *λ*_emission_ = 530 nm) upon enzymatic reduction and subsequent oxidation by ROS, was used [32]. After the preincubation with varying concentrations of ARE solution for 1 h, the cells were treated for 24 h with 1 *μ*g/mL LPS or 10 ng/mL TNF-*α*. The cells were incubated with 5 *μ*M DCFH-DA for 30 min at 37°C and harvested. They were washed with 1 mL FBS-free DMEM two times and resuspended in 1 mL FBS-free DMEM. The intracellular ROS levels were determined by monitoring the fluorescence using a multimode microplate reader (Synergy™ Mx, BioTek Instruments, Winooki, VT, USA).

### 2.14. Western Blotting Analysis

Western blotting analyses were performed to detect iNOS and filaggrin in cellular lysates using anti-iNOS (610332, BD Transduction Laboratories, KY, USA) and anti-filaggrin (SC-30229, Santa Cruz Biotechnology, Dallas, TX, USA) antibodies, respectively. GAPDH, used as an internal loading standard, was detected using anti-GAPDH antibody (LF-PA0212, AbFrontier, Seoul, Korea). After the blotted membrane was incubated with primary antibodies overnight at 4°C, it was reacted with secondary antibody (goat anti-rabbit IgG-pAb-HRP-conjugate; ADI-SAB-300, Enzo Life Sciences, Farmingdale, NY, USA) for 1 h at room temperature and developed using an enhanced West-save up ™ (AbFrontier, Seoul, Korea).

### 2.15. Caspase-14 Activity Assay

Caspase-14 activity in cellular lysates was determined using Ac-WEHD-MCA as a fluorogenic substrate [33]. The reaction mixture (95 *μ*L) consisted of 0.1 M HEPES buffer (pH 7.5), 0.06 M NaCl, 0.01% CHAPS, 5 mM dithiothreitol, 1.3 M sodium citrate, and 10 *μ*M Ac-WEHD-MCA. After cellular lysate (5 *μ*L) was added to the mixture, the total mixture was incubated at room temperature for 30 min. The fluorescent intensity was measured using a multimode microplate reader (Synergy™ Mx, BioTek Instruments, Winooki, VT, USA; *λ*_excitation_ = 355 nm, *λ*_emission_ = 460 nm).

### 2.16. RNA Isolation and Real-Time Quantitative RT-PCR

Total RNA was purified using *FavorPrep™ Tri-RNA Reagent* (FATRR 001, Gentaur, San Jose, CA, USA). cDNA was generated from aliquots of total RNA by reverse transcription using Maxime RT PreMix Kit (25081, iNtRON Biotechnology, Seongnam-si, Korea). Quantitative RT-PCR was performed using TOPreal™ qPCR 2X PreMix (SYBR Green with high ROX, RT501S, Enzynomics, Daejeon, Korea) in a real-time PCR cycler (Rotor-Gene Q, Qiagen, Hilden Germany). The PCR condition used was 95°C penetration for 20 s, 55°C annealing for 20 s, and 72°C extension for 30 s. Primers for human filaggrin mRNA and 18S rRNA were as follows: filaggrin: forward, 5′-AAGGAACTTCTGGAAAAGGAATTTC-3′ and reverse, 5′-TGTGGTCTATATCCAAGTGATCCAT-3′; 18S rRNA: forward, 5′-GCCGCTAGAGGTGAAATTCTTG-3′ and reverse, 5′-CATTCTTGGCAAATGCTTTCG-3′ [34]. The relative amounts of filaggrin mRNA were calculated against 18S rRNA as the invariant control.

### 2.17. Elastase Inhibitory Activity Assay

The elastase inhibitory activity of ARE was examined by measuring a decrease in elastase activity in the presence of ARE. Elastase activity was determined based upon the release of *p*-nitroaniline from *N*-succinyl-(L-Ala)_3_-*p*-nitroanilide used as a substrate [35]. The reaction mixture consisted of 100 *μ*L of 0.2 M Tris buffer (pH 8.0), 100 *μ*L of 0.8 mM *N*-succinyl-(L-Ala)_3_-*p*-nitroanilide, 50 *μ*L of 0.1 U/mL elastase, and 50 *μ*L ARE at the concentrations of 1, 2, 4, and 8 mg/mL. ARE was preincubated with elastase for 20 min at 37°C, and the enzymatic reaction was initiated with the addition of substrate (100 *μ*L). The absorbance at 410 nm was monitored using a microplate reader.

### 2.18. Hyaluronidase Inhibitory Activity Assay

The hyaluronidase inhibitory activity of ARE was examined by measuring a diminishment in hyaluronidase activity in the presence of ARE. The hyaluronidase activity was determined as previously described [36]. The reaction mixture consisted of 10 *μ*L ARE solution, at the concentrations of 0.5, 1, 4, and 8 mg/mL, and 20 *μ*L of 585 U/mL hyaluronidase solution was incubated at 37°C for 10 min and mixed with the equal volume of 0.2 mg/mL HA solution. After 45 min, 240 *μ*L of acidic albumin solution [24 mM sodium acetate, 79 mM acetic acid with 0.1% (*w*/*v*) BSA, pH 3.8 at 25°C] was added to the reaction mixture and allowed to stand for 10 min at room temperature. The absorbance at 600 nm was measured using a microplate reader.

### 2.19. Statistical Analysis

The results were represented as mean ± SD. Differences between experimental groups were analysed using one-way ANOVA followed by post hoc Tukey HSD test for multiple comparisons. A *P* value less than 0.05 was considered statistically significant.

## 3. Results

### 3.1. Total Phenolic Compounds

A variety of phenolic compounds are known to play protective roles against oxidative stress via acting as primary antioxidants or free radical scavengers. The content of total phenolic compounds in ARE was determined to be 38.9 ± 1.7 mg gallic acid equivalent per g extract, indicating that roughly 3.9% of the total constituents in ARE might be phenolic compounds, like gallic acid, on a dry weight basis.

### 3.2. *In Vitro* Antioxidant Potentials

**Figure 1:**
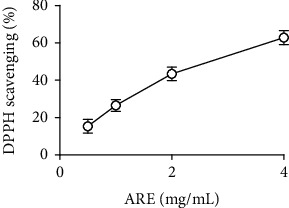
The DPPH radical scavenging activity of ARE (a hot water extract of *Agastache rugosa* leaves). AA, used as a positive control, showed an SC_50_ value of 0.03 mg/mL.

In order to further estimate the total antioxidant activity of ARE, the DPPH radical scavenging assay was conducted. ARE displayed an effective DPPH radical scavenging activity with an SC_50_ of 2.9 mg/mL (Figure 1). AA, used as a positive control, exhibited an SC_50_ of 0.03 mg/mL.

**Figure 2:**
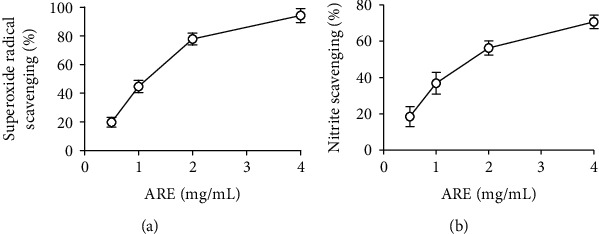
The superoxide radical (a) and nitrite (b) scavenging activities of ARE. AA, used as a positive control, showed the SC_50_ values of 0.5 and 0.06 mg/mL in superoxide radical and nitrite scavenging assays, respectively.

ARE, at the concentrations of 0.5, 1, 2, and 4 mg/ml, was able to scavenge the superoxide radical species, giving rise to the percentage inhibition of 19.8, 44.7, 77.8, and 94.3%, respectively (Figure 2(a)). Its SC_50_ value was 1.4 mg/mL. AA, used as a positive control, exerted an SC_50_ value of 0.5 mg/mL.

As shown in Figure 2(b), ARE exhibited a nitrite scavenging activity. When ARE was used at the concentrations of 0.5, 1, 2, and 4 mg/mL, it could scavenge nitrite ions showing the percentage inhibition of 18.5, 36.9, 56.3, and 70.7%, respectively (Figure 2(b)). Its SC_50_ value was 1.7 mg/mL. AA, used as a positive control, showed an SC_50_ value of 0.06 mg/mL.

These results imply that ARE possesses antiradical, superoxide radical, and nitrite scavenging activities, albeit being relatively lower than the corresponding activities of AA, used as a positive control.

### 3.3. Nontoxicity

**Figure 3:**
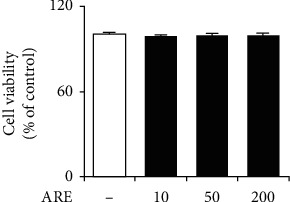
Nontoxic effect of ARE on cellular viability in HaCaT keratinocytes. HaCaT cells were subjected to the fresh medium with the varying concentrations (0, 10, 50, and 200 *μ*g/mL) of ARE for 1 h. The viable cell numbers, represented as the percentage of control, were determined using MTT assay. The experiment was repeated three times.

As shown in Figure 3, ARE displayed no cytotoxicities and gave rise to the similar cellular viabilities, compared with those of the nontreated control. In brief, ARE, at the used concentrations, is not toxic to HaCaT keratinocytes.

### 3.4. Suppression on the LPS-Stimulated NO and ROS Production

**Figure 4:**
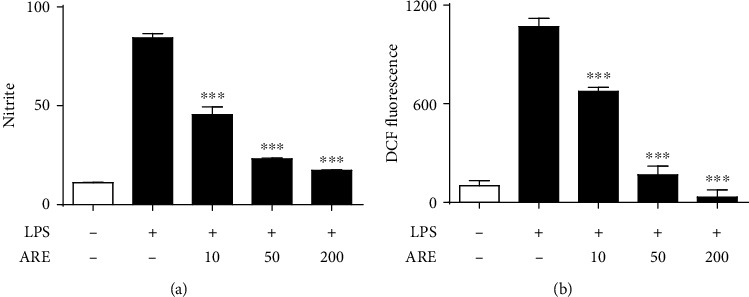
Effects of ARE on the LPS-stimulated elevations of NO (a) and ROS (b) in keratinocytes. After the 5.0 × 10^5^ HaCaT cells were preincubated with the varying concentrations (0, 10, 50, and 200 *μ*g/mL) of ARE, the cells were treated with 1 *μ*g/mL LPS for 24 h. In (a), accumulated nitrite, an index of NO, in conditioned medium was determined based upon Griess reaction. In (b), the ROS level is represented as DCF fluorescence, an arbitrary unit. Each bar shows the mean ± SD of three independent experiments repeated in triplicate. ^∗∗∗^*P* < 0.001 versus the LPS only.

Suppression of NO production is closely linked with an anti-inflammatory action inside living cells. An example is that total flavonoids purified from Radix Glycyrrhiza exert an anti-inflammatory activity via the suppression of iNOS expression in RAW macrophages [37]. When HaCaT keratinocytes were stimulated with LPS, the nitrite content was increased about 8.5-fold (Figure 4(a)). When HaCaT keratinocytes were pretreated with ARE at 10, 50, and 200 *μ*g/mL, the nitrite contents were attenuated to 53.2, 27.9, and 19.8% of those of HaCaT keratinocytes with LPS only (Figure 4(a)). The dose required for half-maximal inhibition (IC_50_) of ARE was 10.8 *μ*g/mL.

ROS, acting as key signaling molecules that play a critical role in inflammatory process, promotes chronic inflammation through the induction of cyclooxygenase-2, inflammatory cytokines (TNF-*α*, interleukin-1, interleukin-6), chemokines (interleukin-8, C-X-C chemokine receptor type 4), and pro-inflammatory transcription factors [38]. In HaCaT keratinocytes, the ROS levels were enhanced to 11.6-fold by LPS (Figure 4(b)). The elevated ROS levels were attenuated to 62.9, 14.7 and 2.6% of those of HaCaT keratinocytes with LPS only by ARE at 10, 50, and 200 *μ*g/mL, respectively (Figure 4(b)). The dose required for half-maximal inhibition (IC_50_) of ARE was 14.8 *μ*g/mL.

Collectively, ARE is able to attenuate the production of both NO and ROS which is elevated in LPS-stimulated HaCaT keratinocytes.

### 3.5. Suppression on the TNF-*α*-Induced NO and ROS Production

**Figure 5:**
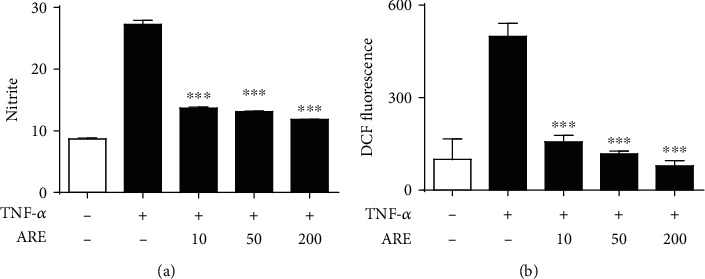
Effects of ARE on the TNF-*α*-stimulated elevations of NO (a) and ROS (b) in keratinocytes. After the 5.0 × 10^5^ HaCaT cells were preincubated with the varying concentrations (0, 10, 50, and 200 *μ*g/mL) of ARE, the cells were treated with 10 ng/mL TNF-*α* for 24 h. In (a), accumulated nitrite, an index of NO, in conditioned medium was determined based upon Griess reaction. In (b), the ROS level is represented as DCF fluorescence, an arbitrary unit. Each bar shows the mean ± SD of three independent experiments repeated in triplicate. ^∗∗∗^*P* < 0.001 versus the LPS only.

In Figure 5, TNF-*α*, a pro-inflammatory cytokine, was similarly used in place of LPS. TNF-*α* could enhance the NO levels to 3.2-fold, compared with those of the nontreated HaCaT keratinocytes (Figure 5(a)). ARE, at 10, 50, and 200 *μ*g/mL, attenuated the TNF-*α*-induced NO levels to 50.4, 47.8, and 42.6%, respectively, giving an IC_50_ value of 9.5 *μ*g/mL (Figure 5(a)). Likewise, the ROS levels were significantly induced by TNF-*α*, and the elevated ROS levels could be attenuated by ARE (Figure 5(b)). As shown in Figure 5(b), ARE, at 10, 50 and 200 *μ*g/mL, was able to attenuate the TNF-*α*-induced ROS levels to 31.7, 22.8, and 14.9%, respectively, which gave rise to an IC_50_ value of 0.4 *μ*g/mL (Figure 5(b)). Taken together, ARE exhibits suppressive effects on the TNF-*α*-induced NO and ROS elevation in HaCaT keratinocytes.

### 3.6. Downregulation of LPS-Stimulated iNOS Production

**Figure 6:**
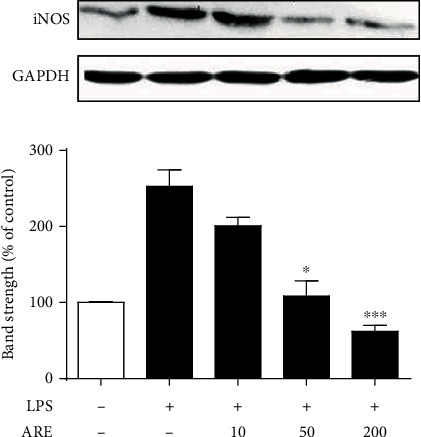
Suppressive effect of ARE on the LPS-stimulated-inducible iNOS production in HaCaT keratinocytes. After the 5.0 × 10^5^ HaCaT cells were preincubated with the varying concentrations (0, 10, 50, and 200 *μ*g/mL) of ARE, the cells were treated with 1 *μ*g/mL LPS for 24 h. iNOS in cellular lysates was detected using western blotting analysis. GAPDH was used as a protein loading control. In the lower panel, the relative band strength, expressed as % of control, was determined with densitometry using the ImageJ software which can be downloaded from the NIH website. ^∗^*P* < 0.05; ^∗∗∗^*P* < 0.001 versus the LPS only.

Effects of ARE on the production of iNOS in the LPS-stimulated HaCaT keratinocytes were reevaluated using Western blotting analysis. LPS enhanced the iNOS protein levels to 2.5-fold in HaCaT keratinocytes (Figure 6). ARE, at 10, 50, and 200 *μ*g/mL, was able to diminish the LPS-induced iNOS production to 80.2, 43.0, and 24.4%, respectively, giving rise to an IC_50_ value of 44.5 *μ*g/mL (Figure 6). This finding confirms that ARE has an attenuating ability on the NO elevation via downregulation of the LPS-stimulated iNOS production in HaCaT keratinocytes.

### 3.7. Upregulation of UV-B-Reduced Filaggrin

**Figure 7:**
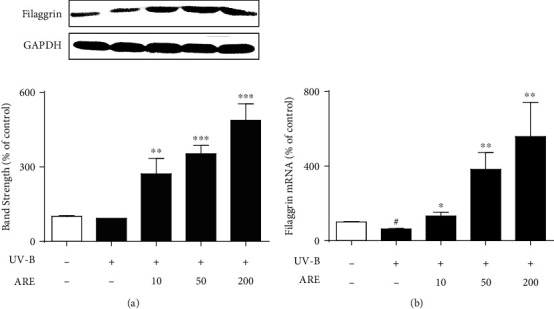
Effects of ARE on the UV-B-reduced filaggrin protein (a) and mRNA (b) levels in keratinocytes. The 5.0 × 10^5^ HaCaT cells were subjected to the varying concentrations (0, 10, 50, and 200 *μ*g/mL) of ARE for 1 h prior to the UV-B irradiation. In (a), the filaggrin proteins in cellular lysates were determined using western blotting analysis. GAPDH was used as a protein loading control. In (b), the filaggrin mRNA levels were determined using quantitative real time RT-PCR analysis. In (a), the relative band strength, expressed as the percentage of control, was determined with densitometry using the ImageJ software that can be downloaded from the NIH website. ^#^*P* < 0.05 versus the nonirradiated control. ^∗^*P* < 0.05; ^∗∗^*P* < 0.01; ^∗∗∗^*P* < 0.001 versus the nontreated control (UV-B irradiation alone).

As shown in Figure 7(a), the UV-B irradiation tended to diminish the filaggrin protein levels in HaCaT keratinocytes. ARE, at 10, 50, and 200 *μ*g/mL, enhanced the UV-B-reduced filaggrin protein levels to 3.3-, 4.3-, and 6.0-fold, respectively, compared to those of the irradiation only (Figure 7(a)). When filaggrin mRNAs were quantitated using quantitative RT-PCR, the UV-B irradiation significantly diminished the filaggrin mRNA levels in HaCaT keratinocytes (Figure 7(b)). ARE, at the concentrations of 10, 50, and 200 *μ*g/mL, could significantly enhance the filaggrin mRNA levels to 2.0-, 6.0-, and 8.8-fold, respectively, compared to those of the irradiation only (Figure 7(b)). Collectively, ARE has an upregulating activity on the expression of filaggrin in the UV-B-irradiated HaCaT keratinocytes.

### 3.8. Upregulation of UV-B-Reduced Caspase-14 Activity

**Figure 8:**
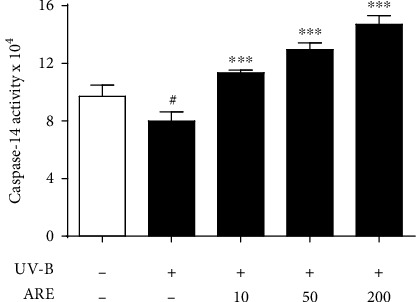
Enhancing effects of ARE on the UV-B-reduced caspase-14 activity levels in HaCaT keratinocytes. The 5.0 × 10^5^ HaCaT cells were subjected to the varying concentrations (0, 10, 50, and 200 *μ*g/mL) of ARE for 1 h prior to the UV-B irradiation. The caspase-14 activity in cellular lysates, expressed as an arbitrary fluorescence unit, was determined using Ac-WEHD-methyl-coumarin amide as a fluorometric substrate. ^#^*P* < 0.05 versus the nonirradiated control. ^∗∗∗^*P* < 0.001 versus the nontreated control (UV-B irradiation alone).

As shown in Figure 8, the UV-B irradiation, at the radiation intensity used, diminished the caspase-14 activity to 80.5% in HaCaT keratinocytes. ARE, at the concentrations of 10, 50, and 200 *μ*g/mL, was able to upregulate the UV-B-reduced caspase-14 activity to 1.4-, 1.6-, and 1.8-fold, respectively, compared to those of the irradiation only (Figure 8). This finding implies that ARE has an upregulating ability on the caspase-14 activity in the UV-B-irradiated HaCaT keratinocytes, which additionally suggests the improving property of ARE on skin barrier function.

### 3.9. Inhibitory Activities on Elastase and Hyaluronidase

**Figure 9:**
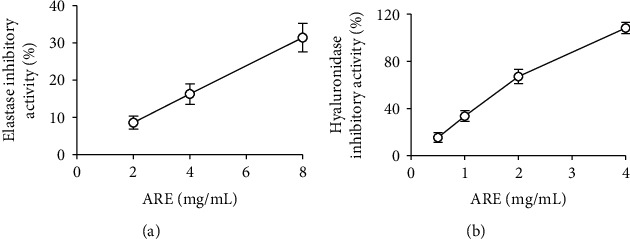
The inhibitory activities of ARE on elastase (a) and hyaluronidase (b) activities. EGCG, used as a positive control in the elastase inhibition assay, showed an IC_50_ value of 1.2 mg/mL, whereas apigenin, used as a positive control in the hyaluonidase inhibition assay, showed an IC_50_ value of 3.5 mg/mL.

When ARE, at the concentrations of 2, 4, and 8 mg/mL, was used in the inhibition assay, it was able to inhibit porcine pancreas elastase activity, showing the percentage inhibition of 8.6, 16.3, and 31.5%, respectively (Figure 9(a)). The IC_50_ value of ARE was estimated to be higher than 8 mg/mL. EGCG, used as a positive control, showed an IC_50_ value of 1.2 mg/mL.

ARE, at the concentrations of 0.5, 1, 2, and 4 mg/mL, exerted an inhibitory activity on hyaluronidase, giving rise to the percentage inhibition of 15.4, 33.6, 67.2, and 108.3%, respectively (Figure 9(b)). Its IC_50_ value was determined to be 1.6 mg/mL. Apigenin, used as a positive control, displayed an IC_50_ value of 3.5 mg/mL.

The inhibition by ARE on *in vitro* elastase and hyaluronidase activities implies its antiwrinkle activity.

## 4. Discussion

Antioxidant-related properties of *A. rugosa* extracts were reported in the aspects of pharmacology. *A. rugos*a leaf extract protects RAW264.7 macrophage cells from hydrogen peroxide-induced injury via the induction of protein kinase G-dependent heme oxygenase-1, which proposes one of action mechanisms of the extract as an antioxidant [39]. *A. rugosa* leaf extract attenuates expression of iNOS and NO production in ROS 17/2.8 cells activated by a mixture of inflammatory cytokines including TNF-*α* and interleukin-1*β* and reduces the cellular toxicity induced by sodium nitroprusside, a nitric oxide donor, suggesting its beneficial role in NO-mediated conditions such as osteoporosis [40]. For the first time, this work demonstrates that ARE [23] possesses skin barrier protective and antiwrinkle properties, through the experiments with HaCaT keratinocytes. Further extensive approaches on the dermatological benefits of *A. rugosa* would be needed to clearly support its topical application to the skin as a skin care remedy as well as prove its use for therapeutic purposes in traditional folk medicine.

Skin barrier-protective function has been regarded as one of crucial targets in the manufacture of functional cosmetics. Barrier function and hydration of psoriatic skins are defective and secondary structure in SC proteins is altered in the involved psoriatic skin [41]. Acute psychosocial and sleep deprivation stress disrupt skin barrier function in women, which results from stress-induced changes in cytokine secretion [42]. Dietary glucosylceramide improves the skin barrier function through the reinforcement of CE formation via transglutaminase expression and involucrin production in the epidermis mediated by sphingoid bases, its metabolites [43]. Topical application of hesperidin, a flavanone glycoside found in orange feel, enhances skin barrier recovery, after acute barrier abrogation, due to stimulation of epidermal proliferation, differentiation as well as lamellar body secretion [44]. In this work, we demonstrate that ARE also possesses a plausible improving activity on skin barrier function through upregulating the UV-B-reduced filaggrin and caspase-14 in HaCaT keratinocytes. In addition to a skin anti-inflammatory activity, the improving properties of ARE on skin barrier function implies its therapeutic usefulness in protecting the human skin under aged and environmentally damaged conditions.

Diverse compounds and mixtures of natural origin have been reported to have antiwrinkle properties based on diverse action mechanisms. Clitocybin A, an isoindolinone isolated from a mycelium extract of the wild Korean mushroom, *Clitocybe aurantiaca*, exhibits an antiwrinkle effect through the enhancement of ROS scavenging and elastase inhibitory activities, and procollagen synthesis in human primary fibroblast-neonatal cells [45]. A leaf ethanol extract of *Aceriphyllum rossii*, a perennial herb endogenous to Korea, contains an antiwrinkle effect through the upregulation of type I procollagen synthesis and the inhibition of collagenase and elastase activities and MMP-1 in human dermal fibroblasts [46]. An aqueous extract of tuna heart exerts antiaging and antiwrinkle effects on human fibroblasts, via attenuating elastase activity and increasing tissue inhibitors of MMP-1 and collagen synthesis [47]. Bark and pod extracts of *Libidibia ferrea*, known as jucẚ possessing potent antioxidant and enzymatic inhibitory activities, were shown to have antiwrinkle and antiwhitening effects via their hyaluronidase, proMMP-2 and tyrosinase inhibitory activities [48]. The antiwrinkle properties of *A. rugosa*, proposed in this work, may be estimated to be based on antioxidant-related mechanism, although the precise mechanism currently remains unsolved.

Throughout this *in vitro* study, we could assess that a hot water extract of *A. rugosa* leaves, named in this work as ARE, have several skin beneficial properties, including barrier improving and antiwrinkle activities, in addition to antioxidant ant anti-inflammatory activities. These findings imply that *A. rugosa* may have potential therapeutic effects on inflammatory skin disorders, such as AD and psoriasis, associated with barrier dysfunction. Its cosmeceutical effect on barrier dysfunction in the aged skins may be another interest, although further studies would be required to verify its feasibility.

## 5. Conclusions

A hot water extract (ARE), prepared from the dried leaves of *Agastache rugosa*, was found to possess both antiradical activity and specific free radical scavenging activities against superoxide radical and nitrite ions. ARE exerted potent suppressive properties on the NO and ROS levels enhanced by LPS or TNF-*α*, and an inhibitory property on the LPS-stimulated iNOS production in HaCaT keratinocytes. It was also capable of upregulating UV-B-reduced filaggrin and caspase-14 in HaCaT keratinocytes. ARE was also identified to contain strong inhibitory activities on elastase and hyaluronidase. Taken together, it contains anti-inflammatory, barrier protective, and antiwrinkle properties, hinting its probable use as a potent skin-healing resource in the manufacture of functional cosmetics.

## Data Availability

The data used to support the findings of this work are available to other researchers from the corresponding author upon request.
